# Two-year weight, risk and health factor outcomes of a weight-reduction intervention programme: Primary prevention for overweight in a multicentre primary healthcare setting

**DOI:** 10.1080/02813432.2020.1753379

**Published:** 2020-05-02

**Authors:** Marie Bräutigam-Ewe, Marie Lydell, Håkan Bergh, Cathrine Hildingh, Amir Baigi, Jörgen Månsson

**Affiliations:** a Department of Public Health and Community Medicine/Primary Health Care, The Sahlgrenska Academy at the University of Gothenburg, Gothenburg, Sweden;; b School of Health and Welfare, Halmstad University, Halmstad, Sweden;; c Research and Development Unit Region of Halland, Sweden

**Keywords:** Obesity, overweight, primary health care, quality of life, sweden, weight-reduction programme

## Abstract

**Objective:** To study the long-term effects of weight reduction, quality of life and sense of coherence in a primary health care (PHC)-based programme with two different intensities.

**Design:** Prospective two-armed randomised intervention.

**Setting:** Three PHC centres in south west of Sweden.

**Subjects:** In total, 289 women and men aged 40-65 years with a BMI of 28-35 were recruited for a two-year weight-reduction programme. Participants were randomized to high-intensity or low-intensity groups. Blood samples, physical measurements and questionnaires were analysed. Participants received cookbooks and dietary lectures. The high-intensity group also received Motivational interviewing (MI), dietary advice on prescription (DAP- advice), a grocery store lecture, a website and weekly e-mails.

**Main outcome measures:** Weight, quality of life, risks and health factors.

**Results:** In total, 182 (64%) participants completed the 2-year follow-up. The total sample reduced their weight by 1 kg (*p* = 0.006). No significant differences regarding weight were found between the groups. Anxiety/depression decreased in EQ5-D (*p* = 0.021), EQ5-D VAS (*p* = 0.002) and SOC (*p* = 0.042). Between the groups, there were significant differences in EQ5-D usual activities (*p* = 0.004), anxiety/depression (*p* = 0.013), pain/discomfort *(p =* 0.041), fruit and vegetables (*p* = 0.005), HLV anxiety (*p* = 0.005), and visits to nurses (*p* = 0.012).

**Conclusion:** The total population lost weight, and the high-intensity and low-intensity programmes did not result in significant differences in terms of weight. The high-intensity programme reported health benefits linked to lower levels of anxiety and depression, increased activity and intake of greens and reduced visits to physicians and nurses.

**Key points**
- Both groups had a consisting weight- reduction after two years.
- High intensity did not lead to a significant difference in weight reduction between the groups.
- The high-intensity group reported more health effects, such as better quality of life, reduced anxiety, and increased greenery intake. It is unknown how much support patients in a weight- reduction programme in PHC require to succeed with weight loss and a healthy lifestyle

## Introduction

Worldwide, the rate of obesity has nearly tripled since 1975. In 2016, more than 1.9 billion adults, 18 years and older, were overweight, and of these, over 650 million were obese [1]. The consequences of this development are increasing rates of cardiovascular diseases, type 2 diabetes, several cancers and psychological disorders (anxiety, depression which are conditions that occur frequently in obese subjects [2]. Since 1980, the occurrence of obesity in Sweden has tripled among adults, and half of Swedes are overweight or obese [3]. Once a patient is obese, weight loss is difficult to achieve and more difficult to maintain [4]. According to a Finnish study that have shown that those who succeed with weight maintenance are less sedentary, are more physically active (one hour per day), consume less alcohol and smoked less [5]. Addressing these issues requires behaviour and lifestyle modifications and the provision of individualised therapy [6].

Primary healthcare (PHC) plays an important role in supporting individuals with lifestyle issues via disease prevention and health promotion activities. There are guidelines focusing on supporting lifestyle changes to prevent unhealthy eating habits and inadequate physical activity [7].

Different methods have been tested within the PHC setting, and motivational interviewing (MI) [8] is a method that is recommended by the National Guidelines for Disease Prevention [7]. PHC could play a more active role in lifestyle change interventions because PHC is the first point of contact with the patient and often involves frequent interaction. Nurses currently provide some support for lifestyle change among patients at risk [9]. According to another study there is not “one size fits all strategy” for successful weight loss maintenance and some individuals require more strategies than others [10]. However more knowledge is needed on how PHC can help and support patients to increase compliance and reinforce diet and behavioural changes over a long period of time and whether more intense support can produce better results.

The main aim of this study was to evaluate the long-term effects of weight reduction and also quality of life and sense of coherence in a primary health care (PHC)-based primary intervention programme. The secondary aim was to compare two subgroups with two different intensities, to each other over time.

## Materials and methods

This study consisted of a prospective two-armed randomized intervention trial.

### Study population

**Figure 1.**
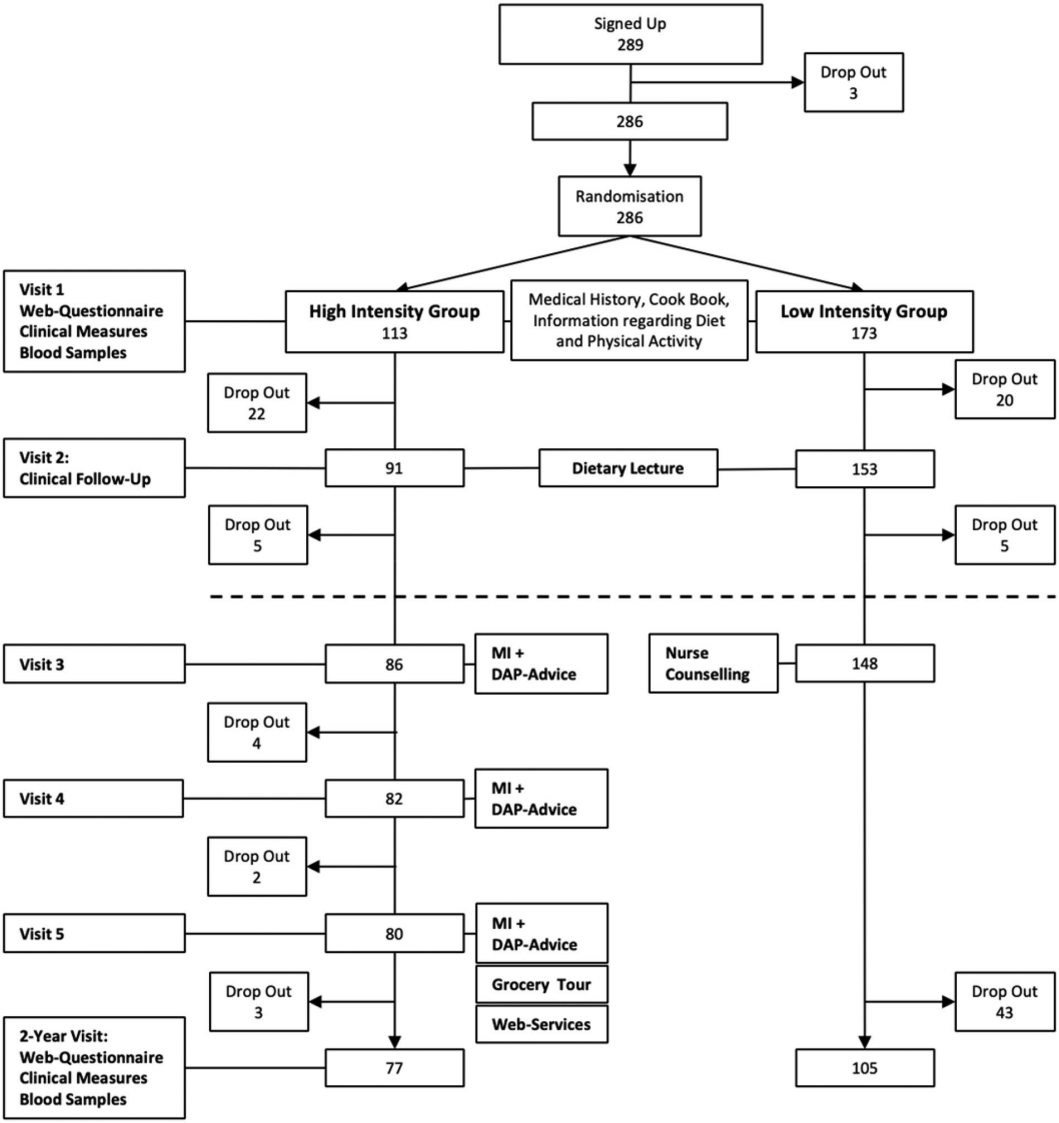
Study flowchart.

The participants included in the study were between 40 and 65 years of age with a body mass index (BMI) of 28–35. Participants were recruited from three different PHC centres in southwest Sweden. The three PHC centres were located in different geographical and socioeconomic areas. Participants were recruited using information in the waiting room of the PHC centres and advertisements in the local newspaper. Patients were excluded if they were undergoing treatment that could be affected by participating in the study, if they had known drug addictions or if they could not understand or produce Swedish in speech or writing. All participants provided written informed consent prior to their inclusion in the programme. A consecutive randomization was performed and because of an expectation of a high dropout rate in the low-intensity group, the randomization was 2:1 in the beginning, which was adjusted to 1:1 when the dropouts were equally distributed (Figure 1) from autumn 2013 to spring 2014; finally, 286 participants were included in the study.

Married couples or friends were included in the same group. The study was blinded in regard to the low- or high-intensity group for participants but not for the nurses. All participants were informed that the study contained two groups with different support. The first and the second visits were carried out in the same way for both groups and involved measurements and blood samples, and the participants also received a link to complete surveys on a computer at home. All procedures were performed with support from the supporting nurse (the nurse who organized the study at each PHC and met all participants other than those in the low-intensity group at visit 3) (Figure 1). The participants received the Nordiet cookbook to read and use until the next visit [11]. The two groups were invited to attend separate dietary lectures by a dietician so that the groups did not influence each other.

After the two visits with the supporting nurse, the low-intensity group met another nurse who worked within the PHC centre, and they had to fill in a dietary form in a traditional way [12]. They received dietary advice from the nurse reflecting care as usual. This procedure was the same for the low-intensity group at all three PHC centres. No more interventions were performed in this group until the follow-up examination after two years when they met the supporting nurse again, and the visit was carried out in the same way as the first visit.

In addition to the low-intensity programme, the high-intensity programme included MI, a grocery store lecture, website communication and weekly e-mails. The participants participated in MI conversations three times about lifestyle habits (diet, physical activity, sleep, stress, alcohol, support, tobacco and mental health), and advice was provided in a dialogue with the patient and included verbal and written individualised lifestyle recommendations from the nurse. The plate model for weight loss was recommended, with a diet composed of approximately 50% vegetables, 25% protein, mostly chicken and fish, and 25% carbohydrates. The focus was not only on the diet but also on how to eat. The participants received DAP-Advice (three postcards with the messages 1. ‘Eat only at the dinner table’; 2. ‘Place your knife and fork on the plate after every mouthful of food’; and 3. ‘Try to regularly eat breakfast, lunch and dinner’) [13] (Figure 1). After two years, both groups were called for a follow-up examination that followed the same procedure as the first visit.

### Data collection

Recruitment occurred between March 2011 and April 2014. The two-year follow-up was completed in April 2016, and the data were collected by means of web-based questionnaires completed by the participants at home. At the first and final visits, participants also had their blood samples and measurements taken. The definition of educational levels was made according to the standards from the Swedish Central Bureau of Statistics (SCB) [14] and the classification consists of six levels of education, with an algorithm defining three levels (short, middle and long).

### Measurements

Height and weight were measured by a nurse at the PHC centre with the participants in a standing position without shoes or outer garments. Height was recorded to the nearest 0.5 cm and weight to the nearest 0.1 kg using a digital scale. BMI was calculated as weight (kg) divided by height squared (m^2^). Waist circumference was measured by the nurse at the level midway between the lower rib margin and the iliac crest. The subjects were asked to breathe out gently during the measurement. The tape was held firmly in a horizontal position.

At baseline and after two years, not fasting blood samples were measured with lipid profiles, and gluco-metabolic status by HbA1c test. Blood pressure was measured by the nurse with the subject in a seated position on the right arm after resting for at least five minutes.

All three support nurses in each PHC centre had two days of training in MI. To improve the knowledge and to standardize the method at each PHC centre, they received further 1-day education in the MI method from a MI teacher. Through conversational exercises, they had to express empathy, positive expectation, listening more than talking themselves, waiting for responses, empowering open questions, reflective listening, dealing with resistance and giving information if needed. This training was performed in a manner that could be feasible with PHC resources in the future. The MI conversations were conducted only in the high-intensity group.

### Questionnaires

#### National health survey (HLV)

The national public health survey includes questions regarding physical and mental health, drug use, healthcare contacts, dental health, lifestyle, socioeconomic conditions, employment, safety, security, intakes of fruit and vegetables and social relations. The questionnaire includes eighty topics. The survey has been conducted annually since 2004, and at the regional level, it is conducted every fourth year [15]. We chose to present two of these topics in this article (anxiety and visits to the PHC). The scale for symptoms was a 3-point scale: no problems, mild problems or severe problems. The questions regarding visits to physicians, nurses or psychologists were as follows: Have you visited one of the following during the last three months? How often? (never, once, several times).

#### Sense of coherence (SOC-13)

Sense of coherence (SOC-13) was measured by the 13-item scale [16]. The scale consists of three dimensions: comprehensibility (five questions), manageability (four questions), and meaningfulness (four questions). The participants were asked to answer the questions on a seven-point scale from 1 (= never) to 7 (= always) with the total score ranging from 13 (low SOC) to 91 (high SOC).

#### EuroQol 5-dimensions (EQ5-D)

QoL was measured using the EQ-5D, which consists of items classified into five dimensions: mobility, self-care, usual activity, pain/discomfort, and anxiety/depression. Each item was answered using one of three responses: no problem, some problems, and extreme problems. The EQ5-D is one standard measurement scale for QoL [17].

The respondent’s response was weighted and calculated as a score value where full health = 1 and death = 0. In this way, both good score and reasonably good score were combined in one and the same dimension [18].

On the thermometer-like scale (EQ VAS), individuals describe how good or bad they feel by marking the scale from 0 to 100 where 0 is the worst possible health and 100 is the best possible health, thus generating the EQ VAS individual self-estimated health condition, defined as the VAS score. In order for the difference in results to be calculated, a change of 0.10 in the score value is required and a change of 10 steps in the barometer.

### Statistical analysis

Descriptive statistics are used to describe the variables and distribution. Dichotomization of the EQ5-D values was based on the EuroQol principle: The value 1 corresponds to complete health and 0 corresponds to a health condition that the population valued to be as bad as being dead [18]. The dichotomization of the variables related to anxiety and the consumption of fruits and vegetables followed the algorithm, which is used by the public health agency of Sweden on a national basis [15]. The question of fruits and vegetables consisted of eight sequential alternatives as follows; three times a day; two times a day; Once a day; 5-6 times per week; 3-4 times a week; 1-2 times a week; once a month, never.

The weighing and dichotomization of the questions took place through a multiplicative synergy impact according to the following formula; (9 = SYSMIS)(1 =  3)(2 = 2)(3 = 1)(4 = 0.8)(5 = 0.5)(6 = 0.2)(7 = 0.07) (ELSE  = SYSMIS) INTO fruit and vegetables.

The issue of anxiety consisted of three alternatives; No problems, some problems, severe problems. The weighing and dichotomization of the questions was carried out as follows; RECODE anxiety (SYSMIS = 0) (ELSE = COPY) INTO anxiety.

IF (anxiety = 1) anxiety = 1 (anxiety). IF (anxiety = 2 OR anxiety = 3) anxiety = 2 (not anxiety). RECODE anxiety (1 = 0) (2 = 1) (0 = SYSMIS) INTO anxiety.

For comparisons between categorical variables, the chi-square and McNemar tests were used. A chi-square test was used in the calculations to test whether the background variable *education* had an effect on some of the study variables. For comparisons between ordinal variables, the Mann-Whitney U and Wilcoxon Rank tests were performed whereas for comparison between quantitative variables, Student’s t-test and the paired t-test were employed. The level of significance was set to 0.05. We assumed a 2 kg weight loss but with a fairly uncertain range since our assumption was based on empirical experience (mean diff: 2 kg; SD = 10). Given this assumption (α = 0.05 and power 1–β = 0.80), approximately 199 individuals for paired comparison, were required. We expect a loss of approximately 25 - 30% during the follow-up (*n* = 80) [19,20].

## Results

A total of 286 participants were included in the study (231 women, 55 men), and of these, 182 (64%) completed the 24 months of follow-up visits: 68% in the high-intensity group and 61% in the low-intensity group (Figure 1). The mean age was 55.7 years (SD 7.1). The baseline weight was 89.1 kg (SD 11.8). Weight data were available from all participants, but 22 (12%) did not complete the questionnaires. Most of the participants (*n* = 182) who completed the programme had a low or medium level of education. There were no significant differences in all participants’ educational levels between high or low scores of EQ5-D, EQ5-D-VAS or the SOC instrument and there were no statistically significant differences between the completers and the drop-outs regarding EQ5-D, EQ5-D-VAS or the SOC instrument linked to the educational level at baseline.

The total study population (both the intervention and non-intervention group) had lost almost one kg of weight (*p* = 0.006). Participants also exhibited decreases in their waist circumference and BMI. Anxiety measured by the EQ-5-D instrument and the VAS score were positively affected (Table 1). There was also an improvement measured by the SOC instrument after two years (Table 1). There were four groups with five participants in each group who came to the grocery tour. The dietary lessons were well attended in both groups. The website was not used to a great extent, but the weekly e-mails were read by most of the participants in the high-intensity group.

**Table 1.** Comparisons between baseline (B) and 2-year follow-up (F) data for the whole study population (*N* = 182).

Baseline – follow-up (2 years)							
	Mean	Mean	%	%	Median	Median	*p* Value
	(B)	(F)	(B)	(F)	(B)	(F)	
**Weight (Kg)**	89.1	88.2					**0.006**
**BMI**	31.4	31.1					**0.011**
**Waist (cm)**	105.0	103.7					**0.038**
HbA1C (mmol/mol)	38.6	38.2					0.091
Cholesterol (mmol/l)	5.7	5.7					0.700
LDL (mmol/l)	3.6	3.8					0.413
HDL (mmol/l)	1.4	1.5					0.055
SYS BT (mmHg)	133.8	133.5					0.771
DIA BT (mmHg)	84.6	83.7					0.164
EQ5D:							
Mobility			79.7	80.8			0.864
Self-care			96.2	98.9			0.063
Usual activities			84.1	89.0			0.064
Pain/discomfort			62.6	61.0			0.775
**Anxiety/depression**			61.5	70.9			**0.021**
**EQ5D-VAS**					75.0	80.0	**0.002**
**SOC**					73.0	74.0	**0.042**
Fruits/vegetables (less)			16.9	15.8			0.980
General health					2.0	2.0	0.483
Anxiety			28.7	27.6			0.824
Visit to physician					2.0	2.0	0.981
Visit to nurse					1.0	1.0	0.676
Visit to psychologist					1.0	1.0	0.119

Both quantitative and qualitative variables were assessed. A paired *t*-test and the McNemar and Wilcoxon Rank tests were utilized.

### Comparison between the two different intervention programmes

There were no weight reduction differences between the high- and low-intensity groups (Table 2). No differences in metabolic markers could be demonstrated. There was a difference in both anxiety/depression, usual activity and pain/discomfort measured by the EQ5-D instrument respectively (Table 2). Anxiety was also affected, as measured by the HLV instrument, which showed an improvement in the high-intensity group and a worsening in the low-intensity group. No significant differences could be seen in the general health between the groups. A higher intake of fruits and vegetables was demonstrated in the high-intensity group. Health care visits to the nurse decreased in the high-intensity group (Table 2). There were no differences after two years between the high-intensity group and the low-intensity group regarding education level in relation to less anxiety or weight loss.

**Table 2.** Comparisons are first made between the two groups at Baseline.

	Baseline			Follow-up			
	High-intensity group	Low-intensity group	*p* Value	High-intensity group	Low-intensity group	Difference	*p* Value
Weight: (kg): mean (SD)	89.5 (11.3)	88.9 (12.3)	0.737	88.4 (11.5)	88.0 (12.3)	0.2	0.712^a^
BMI: (cm) mean (SD)	31.6 (2.1)	31.2 (1.9)	0.179	31.2 (2.4)	31.0 (2.7)	0.2	0.882^a^
Waist: (cm) mean (SD)	105.5 (7.8)	104.0 (9.1)	0.245	103.3 (12.2)	104.0 (9.1)	2.2	0.112^a^
HbA1c: (mmol/mol) mean (SD)	39.1 (6.8)	38.3 (4.8)	0.353	38.8 (6.3)	37.8 (4.6)	−0.2	0.424^a^
Cholesterol:(mmol/l mean (SD)	5.5 (1.1)	5.8 (1.1)	0.071	5.5 (1.1)	5.8 (1.0)	0	0.344^a^
LDL: (mmol/l) mean (SD)	3.5 (1.0)	3.7 (1.0)	0.184	3.7 (3.6)	3.8 (0.9)	−0.1	0.150^a^
HDL: (mmol/l) mean (SD)	1.5 (0.3)	1.5 (0.4)	0.999	1.5 (0.5)	1.5 (0.4)	0	0.548^a^
SYS BT: (mmHg) mean (SD)	134.5 (15.5)	133.2 (16.6)	0.592	133.3 (12.4)	133.6 (15.2)	1.6	0.241^a^
Dia BT: (mmHg) mean (SD)	86.2 (10.5)	83.5 (9.0)	0.064	84.2 (9.9)	83.3 (9.1)	1.8	0.066^a^
EQ5D:							
Mobility (%)	79.2	80	0.895	79.2	81.9	1.9	0.209^b^
Self-care (%)	94.8	97.1	0.428	98.7	99	−2	0.417^b^
**Usual activities (%)**	79.2	87.6	0.127	90.9	87.6	−11.7	**0.004** ^b^
**Pain/discomfort (%)**	64.9	61	0.592	55.8	64.8	12.9	**0.041** ^b^
**Anxiety/depression (%)**	57.1	64.8	0.293	72.7	69.5	−10.9	**0.013** ^b^
EQ5D-VAS: median (IQR)	75.0 (60.0–80.0)	79.0 (70.0–79.9)	0.148	80.0 (63.8–88.5)	80.0 (68.8–90.0)	−4	0.171^c^
SOC: median (IQR)	75.0 (63.0–81.0)	72.0 (63.0–79.5)	0.319	77.0 (61.0–82.0)	72.5 (63.0–81.0)	−1.5	0.216^c^
**Fruits/vegetables (%) (more)**	79	86	0.215	88	81.5	13.5	**0.005** ^b^
General health: median (IQR)	2.0 (2.0–3.0)	2.0 (2.0–3.0)	0.712	2.0 (2.0–3.0)	2.0 (2.0–3.0)	0	0.964^c^
**No Anxiety (%)**	32.8	25.6	0.290	21.3	31.9	17.8	**0.005** ^c^
Visit to physician: median (IQR)	2.0 (2.0–3.0)	2.0 (2.0–2.0)	0.723	2.0 (2.0–2.0)	2.0 (2.0–2.8)	0	0.160^c^
**Visit to nurse: median (IQR)**	2.0 (1.0–2.0)	1.0 (1.0–2.0)	0.001	1.0 (1.0–2.0)	1.5 (1.0–2.0)	1.5	**0.012** ^c^
Visit to psychologist: median (IQR)	1.0 (1.0–1.0)	1.0 (1.0–2.0)	0.988	1.0 (1.0–1.0)	1.0 (1.0–1.0)	0	0.670^c^

Comparisons are then made between the high-intensity group and the low-intensity group. The tests are based on differences between two occasions within groups; baseline and after the intervention (difference:〖〔HIG_(baseline)_ – HIG_(follow up)_〕– 〔LIG_(baseline)_ – LIG_(follow up)_〕〗(HI: *n* = 77; LI: *n* = 105).

^a^Student’s *t*-test;

^b^Chi-square test;

^c^Median test.

### Dropout analysis

A total of 104 participants dropped out (17.6% men). In total, 36% of participants dropped out: 32% in the high-intensity group and 39% in the low-intensity group. The mean age was 52.4 years (SD 7.3), and the baseline weight was 93.2 kg (SD 13.3), compared to 55,7 years and 89,1 Kg respectively in the total group at baseline. Sixty of the 104 participants answered a dropout questionnaire after the study. The reasons for dropping out were life events, too little support, time shortage or obesity surgery. Out of the 60 respondents, 48% had lost weight on their own. There was no difference in education level between dropouts and those who completed the programme.

## Discussion

### Statement of principal findings

The study results showed consistent weight loss in the entire study group after 24 months despite the fact that most people gain weight six months after a weight-reduction programme. The data are limited and the definitions vary across studies, but it appears that approximately ≈20% of overweight individuals experience successful weight loss [5]. However, we could not find any differences regarding weight reduction between the two intensity groups, probably according to rather similar programs and perhaps a low number of participants. The participants’ self-rated health improved, and the score for anxiety/depression was significantly better after the programme. Depression has been shown to be associated with weight gain in a weight maintenance programme in PHC [21]. Positive changes in mood may promote a ‘healthier psychological climate’ that enhances confidence in pursuing weight management behaviours. It is important to have this in mind when helping this group increase HRQOL because people with obesity need long-term support to help them manage their life situation based on their individual needs and personal resources [22].

### Strengths and weaknesses

Strengths of this study are its prospective randomized design and two-year time frame. To obtain stronger results, we should have recruited more participants, especially because the drop-out rate was higher than expected. However, recruitment took longer time than planned which could be explained of a selection of participants with certain features described in an earlier study (23) such as more frequent attenders in health care and they experienced worse health and more stress (23). Drop-outs occurred more frequently than calculated in the power analysis and were more common in intervention programmes for weight-loss reasons [23]. The drop-out analysis showed that they were younger and weighed more than the average of the total group and drop-outs were not only those who had gained weight. No differences were found according to EQ5-D, EQ5-D-VAS, SOC and education between the study group and the drop-outs, which makes it even more difficult to know the reason for drop- out, but this is in line with results from other studies [23].

The nurses who handled the high-intensity group were trained in and used MI conversation. We did not control which conversational strategy was used by the nurses who handled the low-intensity group. This is a weakness of the study, but it reflects care us usual. A weakness of the study was that it could be obvious for the participants which group they were randomized to in the late part of the study when the interventions finished, but the dropouts were not higher as expected in the low intensity group which could explain that they did not realized the different intensities.

Dichotomization of the EQ5-D instrument, like other dichotomizations of variables with ordinal scale, has its limitations. A particularly important point is that the nuance between reasonably good and very good is wiped away, which can be regarded as a simplification of the health situation. Small samples have their limitations. Although the HLV issues were weighted and established as instruments for national studies, there is still a certain likelihood that their application to studies with limited data can produce relatively limited confidentiality in the results.

We did not choose participants with a lower BMI (25-27) since studies have shown that the BMI associated with the lowest all-cause mortality has changed [24]. Therefore, the BMI range was set at 28–35 to include individuals who were suitable for the PHC centres. People with severe obesity (BMI ≥ 40 kg/m^2^) often have more comorbidities and need other treatments, such as gastric bypass [25].

As we have showed in an earlier study, we had recruited participants from geographically and socio-economically different areas, but it turned out that those who applied for the study were more highly educated and mostly women (80%) [26]. This trend can also affect the resources needed in PHC centres because women may need more psychological support and stress management to succeed with weight loss [27].

### Findings in relation to other studies

As in another study in which participants in an obesity programme were given a low-intensity text message compared to a high-intensity text message, we could not show any difference in weight loss between the groups [28]. An earlier study [23] showed that people who participated in this study were more stressed than the general population and that they experienced worse general health than both the overweight and general populations, but after two years of the programme, a significant improvement in SOC for the whole group was demonstrated. According to another study in which SOC was assessed, overweight women seemed to be more vulnerable to a reduced SOC than the general population and required psychological interventions to effectively cope with stress, especially during weight-loss efforts [27].

The high-intensity group had less frequent visits to the nurse in PHC centre. The increased cost of more extensive weight-reduction programmes could be made up for by the reduced visits to PHC centres among these patients. Studies have shown that it is important to be reminded of lifestyle behaviour to succeed with weight reduction. These reminders may occur through phone calls, e-mail and even with a short message service (SMS) [29]. Although our study showed slight weight loss, reminders to continue with a healthy lifestyle are important to prevent comorbidities. The idea of the website was to reduce face-to-face meetings to save costs, but the website was not used as much as was expected. It may not suit this selected group due to a dominance of female participants at the age of fifty who maybe preferred face to face meetings to discuss their problems, as in another study which showed that face-to-face delivery was preferable for weight maintenance [30]. We used e-mail as a reminder to the participants in the study for the high intensity group, but as in another study we could not see any differences in weight loss despite increased support [28].

### Meaning of the study

No single strategy is appropriate for all individuals for successful weight reduction maintenance. Therefore, PHC centres need to offer different solutions with low or high intensities for each individual. Since women mostly seek this type of programme and often report anxiety and depression, it is important to understand that they require more support to succeed with weight reduction.

## Conclusion

The total study sample lost weight, and it did not seem to matter if they participated in the high-intensity or low-intensity programme in terms of weight, however, the high-intensity programme seemed to provide other health benefits linked to less anxiety, less depression, increased usual activity, pain/discomfort, increased intake of greens and fewer visits to nurses at PHC centres. The above benefits of the high intensity programme show obvious health benefits in addition to weight loss.

## Ethical considerations

The Central Ethics Review Board of the University of Stockholm granted permission for the study (No: 29-2010). Prior ethical approval was obtained for the 2015 healthcare intervention study dietary advice on prescription (DAP) (no. 2010/543).
